# Enhancing human learning via spaced repetition optimization

**DOI:** 10.1073/pnas.1815156116

**Published:** 2019-01-22

**Authors:** Behzad Tabibian, Utkarsh Upadhyay, Abir De, Ali Zarezade, Bernhard Schölkopf, Manuel Gomez-Rodriguez

**Affiliations:** ^a^Networks Learning Group, Max Planck Institute for Software Systems, 67663 Kaiserslautern, Germany;; ^b^Empirical Inference Department, Max Planck Institute for Intelligent Systems, 72076 Tübingen, Germany

**Keywords:** memorization, spaced repetition, human learning, marked temporal point processes, stochastic optimal control

## Abstract

Spaced repetition is a technique for efficient memorization which uses repeated review of content following a schedule determined by a spaced repetition algorithm to improve long-term retention. However, current spaced repetition algorithms are simple rule-based heuristics with a few hard-coded parameters. Here, we introduce a flexible representation of spaced repetition using the framework of marked temporal point processes and then address the design of spaced repetition algorithms with provable guarantees as an optimal control problem for stochastic differential equations with jumps. For two well-known human memory models, we show that, if the learner aims to maximize recall probability of the content to be learned subject to a cost on the reviewing frequency, the optimal reviewing schedule is given by the recall probability itself. As a result, we can then develop a simple, scalable online spaced repetition algorithm, MEMORIZE, to determine the optimal reviewing times. We perform a large-scale natural experiment using data from Duolingo, a popular language-learning online platform, and show that learners who follow a reviewing schedule determined by our algorithm memorize more effectively than learners who follow alternative schedules determined by several heuristics.

## Significance

Understanding human memory has been a long-standing problem in various scientific disciplines. Early works focused on characterizing human memory using small-scale controlled experiments and these empirical studies later motivated the design of spaced repetition algorithms for efficient memorization. However, current spaced repetition algorithms are rule-based heuristics with hard-coded parameters, which do not leverage the automated fine-grained monitoring and greater degree of control offered by modern online learning platforms. In this work, we develop a computational framework to derive optimal spaced repetition algorithms, specially designed to adapt to the learners’ performance. A large-scale natural experiment using data from a popular language-learning online platform provides empirical evidence that the spaced repetition algorithms derived using our framework are significantly superior to alternatives.

Our ability to remember a piece of information depends critically on the number of times we have reviewed it, the temporal distribution of the reviews, and the time elapsed since the last review, as first shown by a seminal study by Ebbinghaus (1). The effect of these two factors has been extensively investigated in the experimental psychology literature (2, 3), particularly in second language acquisition research (4–7). Moreover, these empirical studies have motivated the use of flashcards, small pieces of information a learner repeatedly reviews following a schedule determined by a spaced repetition algorithm (8), whose goal is to ensure that learners spend more (less) time working on forgotten (recalled) information.

The task of designing spaced repetition algorithms has a rich history, starting with the Leitner system (9). More recently, several works (10, 11) have proposed heuristic algorithms that schedule reviews just as the learner is about to forget an item, i.e., when the probability of recall, as given by a memory model of choice (1, 12), falls below a threshold. An orthogonal line of research (7, 13) has pursued locally optimal scheduling by identifying which item would benefit the most from a review given a fixed reviewing time. In doing so, the researchers have also proposed heuristic algorithms that decide which item to review by greedily selecting the item which is closest to its maximum learning rate.

In recent years, spaced repetition software and online platforms such as Mnemosyne (mnemosyne-proj.org), Synap (www.synap.ac), and Duolingo (www.duolingo.com) have become increasingly popular, often replacing the use of physical flashcards. The promise of these pieces of software and online platforms is that automated fine-grained monitoring and greater degree of control will result in more effective spaced repetition algorithms. However, most of the above spaced repetition algorithms are simple rule-based heuristics with a few hard-coded parameters (8), which are unable to fulfill this promise—adaptive, data-driven algorithms with provable guarantees have been largely missing until very recently (14, 15). Among these recent notable exceptions, the work most closely related to ours is by Reddy et al. (15), who proposed a queueing network model for a particular spaced repetition method—the Leitner system (9) for reviewing flashcards—and then developed a heuristic approximation algorithm for scheduling reviews. However, their heuristic does not have provable guarantees, it does not adapt to the learner’s performance over time, and it is specifically designed for the Leitner systems.

In this work, we develop a computational framework to derive optimal spaced repetition algorithms, specially designed to adapt to the learner’s performance, as continuously monitored by modern spaced repetition software and online learning platforms. More specifically, we first introduce a flexible representation of spaced repetition using the framework of marked temporal point processes (16). For several well-known human memory models (1, 12, 17–19), we use this presentation to express the dynamics of a learner’s forgetting rates and recall probabilities for the content to be learned by means of a set of stochastic differential equations (SDEs) with jumps. Then, we can find the optimal reviewing schedule for spaced repetition by solving a stochastic optimal control problem for SDEs with jumps (20–23). In doing so, we need to introduce a proof technique to find a solution to the so-called Hamilton–Jacobi–Bellman (HJB) equation (*SI Appendix*, sections 3 and 4), which is of independent interest.

For two well-known memory models, we show that, if the learner aims to maximize recall probability of the content to be learned subject to a cost on the reviewing frequency, the solution uncovers a linear relationship with a negative slope between the optimal rate of reviewing, or reviewing intensity, and the recall probability of the content to be learned. As a consequence, we can develop a simple, scalable online spaced repetition algorithm, which we name MEMORIZE, to determine the optimal reviewing times. Finally, we perform a large-scale natural experiment using data from Duolingo, a popular language-learning online platform, and show that learners who follow a reviewing schedule determined by our algorithm memorize more effectively than learners who follow alternative schedules determined by several heuristics. To facilitate research in this area, we are releasing an open-source implementation of our algorithm (24).

## Modeling Framework of Spaced Repetition.

Our framework is agnostic to the particular choice of memory model—it provides a set of techniques to find reviewing schedules that are optimal under a memory model. Here, for ease of exposition, we showcase our framework for one well-known memory model from the psychology literature, the exponential forgetting curve model with binary recalls (1, 17), and use (a variant of) a recent machine-learning method, half-life regression (25), to estimate the effect of the reviews on the parameters of such model. [In *SI Appendix*, sections 6 and 7, we apply our framework to other two popular memory models, the power-law forgetting curve model (18, 19) and the multiscale context model (MCM) (12).]

More specifically, given a learner who wants to memorize a set of items I using spaced repetition, i.e., repeated, spaced review of the items, we represent each reviewing event as a triplet

$$e\;≔\;\left(\underset{\overset{↑}{\text{item}}}{i},\;\overset{\underset{↓}{\text{time}}}{t},\;\underset{\overset{↑}{\text{recall}}}{r}\right),$$

which means that the learner reviewed item i∈I at time t and either recalled it (r=1) or forgot it (r=0). Here, note that each reviewing event includes the outcome of a test (i.e., a recall) since, in most spaced repetition software and online platforms such as Mnemosyne, Synap, and Duolingo, the learner is tested in each review, following the seminal work of Reidiger and Karpicke (26).

Given the above representation, we model the probability that the learner recalls (forgets) item i at time t using the exponential forgetting curve model; i.e.,

$$m_{i}\left(t\right)≔P\left(r\right)=\exp\left(-n_{i}\left(t\right)\left(t-t_{r}\right)\right),$$ [1]

where $t_{r}$ is the time of the last review and $n_{i}\left(t\right)\in R^{+}$ is the forgetting rate at time t, which may depend on many factors, e.g., item difficulty and/or number of previous (un)successful recalls of the item. [Previous work often uses the inverse of the forgetting rate, referred to as memory strength or half-life, $s\left(t\right)=n^{-1}\left(t\right)$ (15, 25). However, it is more tractable for us to work in terms of forgetting rates.] Then, we keep track of the reviewing times using a multidimensional counting process N(t), in which the ith entry, $N_{i}\left(t\right)$, counts the number of times the learner has reviewed item i up to time t. Following the literature on temporal point processes (16), we characterize these counting processes using their corresponding intensities u(t), i.e., E[dN(t)]=u(t)dt, and think of the recall r as their binary marks. Moreover, every time that a learner reviews an item, the recall r has been experimentally shown to have an effect on the forgetting rate of the item (3, 15, 25). Here, we estimate such an effect using half-life regression (25), which implicitly assumes that recalls of an item i during a review have a multiplicative effect on the forgetting rate $n_{i}\left(t\right)$—a successful recall at time $t_{r}$ changes the forgetting rate by $\left(1-\alpha_{i}\right)$, i.e., $n_{i}\left(t\right)=\left(1-\alpha_{i}\right)n_{i}\left(t_{r}\right)$, $\alpha_{i}\leq 1$, while an unsuccessful recall changes the forgetting rate by $\left(1+\beta_{i}\right)$, i.e., $n_{i}\left(t\right)=\left(1+\beta_{i}\right)n_{i}\left(t_{r}\right)$, $\beta_{i}\geq 0$. In this context, the initial forgetting rate, $n_{i}\left(0\right)$, captures the difficulty of the item, with more difficult items having higher initial forgetting rates compared with easier items, and the parameters $\alpha_{i}$, $\beta_{i}$, and $n_{i}\left(0\right)$ are estimated using real data (refer to *SI Appendix*, section 8 for more details).

Before we proceed farther, we acknowledge that several laboratory studies (6, 27) have provided empirical evidence that the retention rate follows an inverted U shape, i.e., mass practice does not improve the forgetting rate—if an item is in a learner’s short-term memory when the review happens, the long-term retention does not improve. Thus, one could argue for time-varying parameters $\alpha_{i}\left(t\right)$ and $\beta_{i}\left(t\right)$ in our framework. However, there are several reasons that prevent us from that: (*i*) The derivation of an optimal reviewing schedule under time-varying parameters becomes very challenging; (*ii*) for the reviewing sequences in our Duolingo dataset, allowing for time-varying $\alpha_{i}$ and $\beta_{i}$ in our modeling framework does not lead to more accurate recall predictions (*SI Appendix*, section 9); and (*iii*) several popular spaced repetition heuristics, such as the Leitner system with exponential spacing and SuperMemo, have achieved reasonable success in practice despite implicitly assuming constant $\alpha_{i}$ and $\beta_{i}$. [The Leitner system with exponential spacing can be explicitly cast using our formulation with particular choices of $\alpha_{i}$ and $\beta_{i}$ and the same initial forgetting rate, $n_{i}\left(0\right)=n\left(0\right)$, for all items (*SI Appendix*, section 11).] That being said, it would be an interesting venue for future work to derive optimal reviewing schedules under time-varying parameters.

Next, we express the dynamics of the forgetting rate $n_{i}\left(t\right)$ and the recall probability $m_{i}\left(t\right)$ for each item i∈I using SDEs with jumps. This is very useful for the design of our spaced repetition algorithm using stochastic optimal control. More specifically, the dynamics of the forgetting rate $n_{i}\left(t\right)$ are readily given by

$$dn_{i}\left(t\right)=-\alpha_{i}n_{i}\left(t\right)r_{i}\left(t\right)dN_{i}\left(t\right)+\beta_{i}n_{i}\left(t\right)\left(1-r_{i}\left(t\right)\right)dN_{i}\left(t\right),$$ [2]

where $N_{i}\left(t\right)$ is the corresponding counting process and $r_{i}\left(t\right)\in \left\{0,1\right\}$ indicates whether item i has been successfully recalled at time t. Similarly, the dynamics of the recall probability $m_{i}\left(t\right)$ are given by *Proposition 1* (proved in *SI Appendix*, section 1):

## Proposition 1.

*Given an item* i∈I *with reviewing intensity* $u_{i}\left(t\right)$, *the recall probability* $m_{i}\left(t\right)$, *defined by* *Eq*. **1**, *is a Markov process whose dynamics can be defined by the following SDE with jumps*,

$$dm_{i}\left(t\right)=-n_{i}\left(t\right)m_{i}\left(t\right)dt+\left(1-m_{i}\left(t\right)\right)dN_{i}\left(t\right),$$ [3]

*where* $N_{i}\left(t\right)$ *is the counting process associated to the reviewing intensity* $u_{i}\left(t\right)$†.

Finally, given a set of items I, we cast the design of a spaced repetition algorithm as the search of the optimal item reviewing intensities $u\left(t\right)={\left[u_{i}\left(t\right)\right]}_{i\in I}$ that minimize the expected value of a particular (convex) loss function ℓ(m(t),n(t),u(t)) of the recall probability of the items, $m\left(t\right)={\left[m_{i}\left(t\right)\right]}_{i\in I}$; the forgetting rates, $n\left(t\right)={\left[n_{i}\left(t\right)\right]}_{i\in I}$; and the intensities themselves, u(t); over a time window $\left(t_{0},t_{f}\right]$; i.e.,

$$\begin{array}{ll} \underset{u\left(t_{0},t_{f}\right]}{\text{minimize}} & \;E\left[\phi \left(m\left(t_{f}\right),n\left(t_{f}\right)\right)+\int_{t_{0}}^{t_{f}}\ell \left(m\left(\tau \right),n\left(\tau \right),u\left(\tau \right)\right)d\tau \right]\\ \text{subject to} & \;u\left(t\right)\geq 0\forall t\in \left(t_{0},t_{f}\right), \end{array}$$ [4]

where $u\left(t_{0},t_{f}\right]$ denotes the item reviewing intensities from $t_{0}$ to $t_{f}$, the expectation is taken over all possible realizations of the associated counting processes and (item) recalls, the loss function is nonincreasing (nondecreasing) with respect to the recall probabilities (forgetting rates and intensities) so that it rewards long-lasting learning while limiting the number of item reviews, and $\phi \left(m\left(t_{f}\right),n\left(t_{f}\right)\right)$ is an arbitrary penalty function. [The penalty function $\phi \left(m\left(t_{f}\right),n\left(t_{f}\right)\right)$ is necessary to derive the optimal reviewing intensities $u^{*}\left(t\right)$.] Here, note that the forgetting rates n(t) and recall probabilities m(t), as defined by Eqs. **2** and **3**, depend on the reviewing intensities u(t) we aim to optimize since E[dN(t)]=u(t)dt.

## The MEMORIZE Algorithm.

The spaced repetition problem, as defined by Eq. **4**, can be tackled from the perspective of stochastic optimal control of jump SDEs (20). Here, we first derive a solution to the problem considering only one item, provide an efficient practical implementation of the solution, and then generalize it to the case of multiple items.

Given an item i, we can write the spaced repetition problem, i.e., Eq. **4**, for it with reviewing intensity $u_{i}\left(t\right)=u\left(t\right)$ and associated counting process $N_{i}\left(t\right)=N\left(t\right)$, recall outcome $r_{i}\left(t\right)=r\left(t\right)$, recall probability $m_{i}\left(t\right)=m\left(t\right)$, and forgetting rate $n_{i}\left(t\right)=n\left(t\right)$. Further, using Eqs. **2** and **3**, we can define the forgetting rate n(t) and recall probability m(t) by the following two coupled jump SDEs,

$$\begin{array}{c} dn\left(t\right)=-\alpha n\left(t\right)r\left(t\right)dN\left(t\right)+\beta n\left(t\right)\left(1-r\left(t\right)\right)dN\left(t\right)\\ dm\left(t\right)=-n\left(t\right)m\left(t\right)dt+\left(1-m\left(t\right)\right)dN\left(t\right) \end{array}$$

with initial conditions $n\left(t_{0}\right)=n_{0}$ and $m\left(t_{0}\right)=m_{0}$.

Next, we define an optimal cost-to-go function J for the above problem, use Bellman’s principle of optimality to derive the corresponding HJB equation (28), and exploit the unique structure of the HJB equation to find the optimal solution to the problem.

## 

### Definition 2:

*The optimal cost-to-go* J(m(t),n(t),t) *is defined as the minimum of the expected value of the cost of going from state* (m(t),n(t)) *at time* t *to the final state at time* $t_{f}$:

$$\begin{array}{l} J=\underset{u\left(t,t_{f}\right]}{\min}E_{{\left.\left(N\left(s\right),r\left(s\right)\right)\right|}_{s=t}^{s=t_{f}}}\left[\phi \left(m\left(t_{f}\right),n\left(t_{f}\right)\right)\right.+\left.\int_{t}^{t_{f}}\ell \left(m\left(\tau \right),u\left(\tau \right)\right)d\tau \right]. \end{array}$$ [5]

Now, we use Bellman’s principle of optimality, which the above definition allows, to break the problem into smaller subproblems. [Bellman’s principle of optimality readily follows using the Markov property of the recall probability m(t) and forgetting rate n(t).] With dJ(m(t),n(t),t)=J(m(t+dt),n(t+dt),t+dt)−J(m(t),n(t),t), we can, hence, rewrite Eq. **5** as

$$\begin{array}{c} J\left(m\left(t\right),n\left(t\right),t\right)=\underset{u\left(t,t+dt\right]}{\text{min}}E\left[J\left(m\left(t+dt\right),n\left(t+dt\right),t+dt\right)\right]+\ell \left(m\left(t\right),n\left(t\right),u\left(t\right)\right)dt \end{array}$$

$$0=\underset{u\left(t,t+dt\right]}{\text{min}}E\left[dJ\left(m\left(t\right),n\left(t\right),t\right)\right]+\ell \left(m\left(t\right),n\left(t\right),u\left(t\right)\right)dt.$$ [6]

Then, to derive the HJB equation, we differentiate J with respect to time t, m(t), and n(t) using *SI Appendix*, section 2, *Lemma 1*:

$$\begin{array}{ll} 0 & =J_{t}\left(m,n,t\right)-nmJ_{m}\left(m,n,t\right)\\ & \;+\underset{u\left(t,t+dt\right]}{\text{min}}\left\{\ell \left(m,n,u\right)\left[J\left(1,\left(1-\alpha \right)n,t\right)m\right.\right.\\ & \;+\left.\left.J\left(1,\left(1+\beta \right)n,t\right)\left(1-m\right)-J\left(m,n,t\right)\right]u\left(t\right)\right\}. \end{array}$$ [7]

To solve the above equation, we need to define the loss ℓ. Following the literature on stochastic optimal control (28), we consider the following quadratic form, which is nonincreasing (nondecreasing) with respect to the recall probabilities (intensities) so that it rewards learning while limiting the number of item reviews,

$$\ell \left(m\left(t\right),n\left(t\right),u\left(t\right)\right)=\frac{1}{2}{\left(1-m\left(t\right)\right)}^{2}+\frac{1}{2}qu^{2}\left(t\right),$$ [8]

where q is a given parameter, which trades off recall probability and number of item reviews—the higher its value, the lower the number of reviews. Note that this particular choice of loss function does not directly place a hard constraint on number of reviews; instead, it limits the number of reviews by penalizing high reviewing intensities. (Given a desired level of practice, the value of the parameter q can be easily found by simulation since the average number of reviews decreases monotonically with respect to q.)

Under these definitions, we can find the relationship between the optimal intensity and the optimal cost by taking the derivative with respect to u(t) in Eq. **7**:

$$\begin{array}{l} u^{*}\left(t\right)=q^{-1}{\left[J\left(m\left(t\right),n\left(t\right),t\right)-J\left(1,\left(1-\alpha \right)n\left(t\right),t\right)m\left(t\right)-J\left(1,\left(1+\beta \right)n\left(t\right),t\right)\left(1-m\left(t\right)\right)\right]}_{+}. \end{array}$$

Finally, we plug the above equation into Eq. **7** and find that the optimal cost-to-go J needs to satisfy the following nonlinear differential equation:

$$\begin{array}{ccc} 0 & = & J_{t}\left(m\left(t\right),n\left(t\right),t\right)-n\left(t\right)m\left(t\right)J_{m}\left(m\left(t\right),n\left(t\right),t\right)\\ & & +\frac{1}{2}{\left(1-m\left(t\right)\right)}^{2}-\frac{1}{2}q^{-1}\left[J\left(m\left(t\right),n\left(t\right),t\right)\right.\\ & & -J\left(1,\left(1-\alpha \right)n\left(t\right),t\right)m\left(t\right)\\ & & -{\left.J\left(1,\left(1+\beta \right)n\left(t\right),t\right)\left(1-m\left(t\right)\right)\right]}_{+}^{2}. \end{array}$$

To continue farther, we rely on a technical lemma (*SI Appendix*, section 3, *Lemma 2*), which derives the optimal cost-to-go J for a parameterized family of losses ℓ. Using *SI Appendix*, section 3, *Lemma 2*, the optimal reviewing intensity is readily given by *Theorem 3* (proved in *SI Appendix*, section 4):

## 

### Theorem 3.

*Given a single item*, *the optimal reviewing intensity for the spaced repetition problem*, *defined by* *Eq*. **4**, *under quadratic loss*, *defined by* *Eq*. **8**, *is given by*

$$u^{*}\left(t\right)=q^{-1/2}\left(1-m\left(t\right)\right).$$ [9]

Note that the optimal intensity depends only on the recall probability, whose dynamics are given by Eqs. **2** and **3**, and thus allows for a very efficient procedure to sample reviewing times, which we name MEMORIZE. Algorithm 1 provides a pseudocode implementation of MEMORIZE. Within the algorithm, Sample(u(t)) samples from an inhomogeneous Poisson process with intensity u(t) and it returns the sampled time and ReviewItem(t′) returns the recall outcome r of an item reviewed at time t′, where r=1 indicates the item was recalled successfully and r=0 indicates it was not recalled. Moreover, note that t denotes a (time) parameter, s and t′ denote specific (time) values, and we sample from an inhomogeneous Poisson process using a standard thinning algorithm (29). [In some practical deployments, one may want to discretize the optimal intensity u(t) and, e.g., “at top of each hour, decide whether to do a review or not.”]

**Table:** 

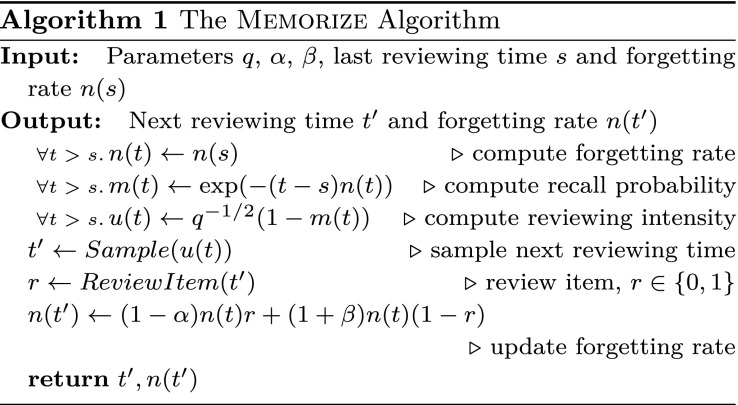

Given a set of multiple items I with reviewing intensities u(t) and associated counting processes N(t), recall outcomes r(t), recall probabilities m(t), and forgetting rates n(t), we can solve the spaced repetition problem defined by Eq. **4** similarly as in the case of a single item. More specifically, consider the following quadratic form for the loss ℓ,

$$\ell \left(m\left(t\right),n\left(t\right),u\left(t\right)\right)=\frac{1}{2}\underset{i\in I}{\sum }{\left(1-m_{i}\left(t\right)\right)}^{2}+\frac{1}{2}\underset{i\in I}{\sum }q_{i}u_{i}^{2}\left(t\right),$$

where ${\left\{q_{i}\right\}}_{i\in I}$ are given parameters, which trade off recall probability and number of item reviews and may favor the learning of one item over another. Then, one can exploit the independence among items assumption to derive the optimal reviewing intensity for each item, proceeding similarly as in the case of a single item:

### Theorem 4.

*Given a set of items* I, *the optimal reviewing intensity for each item* i∈I *in the spaced repetition problem*, *defined by* *Eq*. **4**, *under quadratic loss is given by*

$$u_{i}^{*}\left(t\right)=q_{i}^{-1/2}\left(1-m_{i}\left(t\right)\right).$$ [10]

Finally, note that we can easily sample item reviewing times simply by running |I| instances of MEMORIZE, one per item.

### Experimental Design.

We use data gathered from Duolingo, a popular language-learning online platform (the dataset is available at https://github.com/duolingo/halflife-regression), to validate our algorithm MEMORIZE. (Refer to *SI Appendix*, section 5 for an experimental validation of our algorithm using synthetic data, whose goal is analyzing it under a controlled setting using metrics and baselines that we cannot compute in the real data we have access to.) This dataset consists of ∼12 million sessions of study, involving ∼5.3 million unique (user, word) pairs, which we denote by D, collected over the period of 2 wk. In a single session, a user answers multiple questions, each of which contains multiple words. (Refer to *SI Appendix*, section 12 for additional details on the Duolingo dataset.) Each word maps to an item i and the fraction of correct recalls of sentences containing a word i in the session is used as an empirical estimate of its recall probability $\hat{m}\left(t\right)$ at the time of the session t, as in previous work (25). If a word is recalled perfectly during a session, then it is considered a successful recall, i.e., $r_{i}\left(t\right)=1$, and otherwise it is considered an unsuccessful recall, i.e., $r_{i}\left(t\right)=0$. Since we can expect the estimation of the model parameters to be accurate only for users and items with enough numbers of reviewing events, we consider only users with at least 30 reviewing events and words that were reviewed at least 30 times. After this preprocessing step, our dataset consists of ∼5.2 million unique (user, word) pairs.

We compare the performance of our method with two baselines: (*i*) a uniform reviewing schedule, which sends item(s) for review at a constant rate μ, and (*ii*) a threshold-based reviewing schedule, which increases the reviewing intensity of an item by $c\exp\left(\left(t-s\right)/\zeta \right)$ at time s, when its recall probability reaches a threshold $m^{th}$. The threshold baseline is similar to the heuristics proposed by previous work (10, 11, 30), which schedule reviews just as the learner is about to forget an item. We do not compare with the algorithm proposed by Reddy et al. (15) because, as it is specially designed for the Leitner system, it assumes a discrete set of forgetting rate values and, as a consequence, is not applicable to our (more general) setting.

**Fig. 1.**
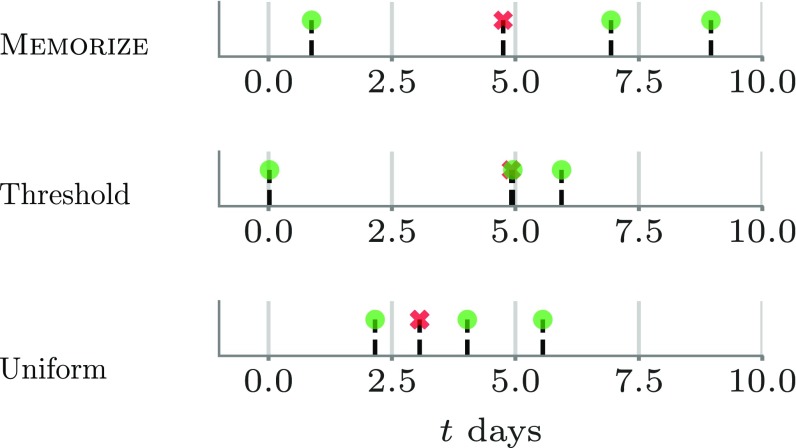
Examples of (user, item) pairs whose corresponding reviewing times have high likelihood under MEMORIZE (*Top*), threshold-based reviewing schedule (*Middle*), and uniform reviewing schedule (*Bottom*). In every plot, each candlestick corresponds to a reviewing event with a green circle (red cross) if the recall was successful (unsuccessful), and time t=0 corresponds to the first time the user is exposed to the item in our dataset, which may or may not correspond with the first reviewing event. The pairs whose reviewing times follow more closely MEMORIZE or the threshold-based schedule tend to increase the time interval between reviews every time a recall is successful while, in contrast, the uniform reviewing schedule does not. MEMORIZE tends to space the reviews more than the threshold-based schedule, achieving the same recall pattern with less effort.

Although we cannot make actual interventions to evaluate the performance of each method, the following insight allows for a large-scale natural experiment: Duolingo uses hand-tuned spaced repetition algorithms, which propose reviewing times to the users; however, users often do not perform reviews exactly at the recommended times, and thus schedules for some (user, item) pairs will be closer to uniform than threshold or MEMORIZE and vice versa, as shown in Fig. 1. As a consequence, we are able to assign each (user, item) pair to a treatment group (i.e., MEMORIZE) or a control group (i.e., uniform or threshold). More in detail, we leverage this insight to design a robust evaluation procedure which relies on (*i*) likelihood comparisons to determine how closely a user followed a particular reviewing schedule during all reviews but the last in a reviewing sequence, i.e., $e_{1},\ldots ,e_{n-1}$ in a sequence with n reviews, and (*ii*) a quality metric, empirical forgetting rate $\hat{n}$, which can be estimated using only the last review $e_{n}$ (and the retention interval $t_{n}-t_{n-1}$) of each reviewing sequence and does not depend on the particular choice of memory model. Refer to *Materials and Methods* for more details on our evaluation procedure. [Note that our goal is to evaluate how well different reviewing schedule spaces the reviews—our objective is not to evaluate the predictive power of the underlying memory models; we are relying on previous work for that (18, 25). However, for completeness, we provide a series of benchmarks and evaluations for the memory models we used in this work in *SI Appendix*, section 8.]

## Results

**Fig. 2.**
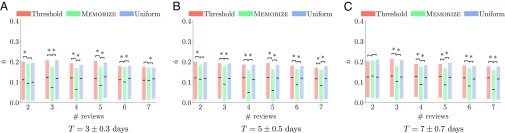
(*A*–*C*) Average empirical forgetting rate for the top 25% of pairs in terms of likelihood for MEMORIZE, the uniform reviewing schedule, and the threshold-based reviewing schedule for sequences with different numbers of reviews n and different training periods $T=t_{n-1}-t_{1}$. Boxes indicate 25% and 75% quantiles and solid lines indicate median values, where lower values indicate better performance. MEMORIZE offers a competitive advantage with respect to the uniform and the threshold-based baselines and, as the training period increases, the number of reviews under which MEMORIZE achieves the greatest competitive advantage increases. For each distinct number of reviews and training periods, * indicates a statistically significant difference (Mann–Whitney *U* test; *P*-value < 0.05) between MEMORIZE vs. threshold and MEMORIZE vs. uniform scheduling.

We first group (user, item) pairs by their number of reviews n and their training period, i.e., $t_{n-1}-t_{1}$. Then, for each recall pattern, we create the treatment (MEMORIZE) and control (uniform and threshold) groups and, for every reviewing sequence in each group, compute its empirical forgetting rate. Fig. 2 summarizes the results for sequences with up to seven reviews since the beginning of the observation window for three distinct training periods. The results show that MEMORIZE offers a competitive advantage with respect to the uniform- and threshold-based baselines and, as the training period increases, the number of reviews under which MEMORIZE achieves the greatest competitive advantage increases. Here, we can rule out that this advantage is a consequence of selection bias due to the item difficulty (*SI Appendix*, section 13).

**Fig. 3.**
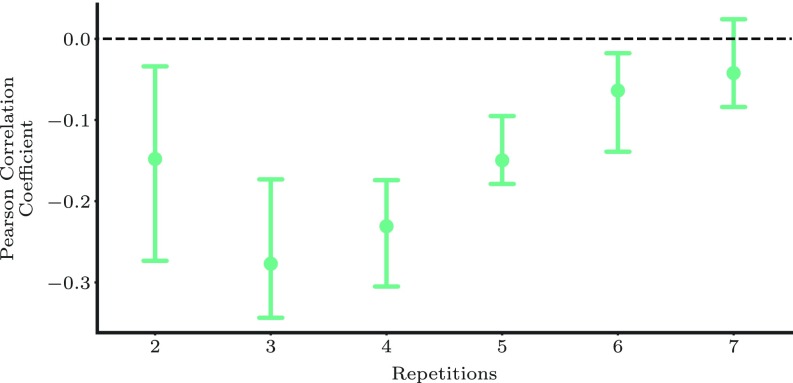
Pearson correlation coefficient between the log-likelihood of the top and bottom 50% of reviewing sequences of a learner under MEMORIZE and its associated empirical forgetting rate. The circles indicate median values and the bars indicate standard error. Lower correlation values correspond to greater gains due to MEMORIZE. To ensure reliable estimation, we considered learners with at least 70 reviewing sequences with a training period T=8±3.2 d. There were 322 of such learners.

Next, we go a step farther and verify that, whenever a specific learner follows MEMORIZE more closely, her performance is superior. More specifically, for each learner with at least 70 reviewing sequences with a training period T=8±3.2 d, we select the top and bottom 50% of reviewing sequences in terms of log-likelihood under MEMORIZE and compute the Pearson correlation coefficient between the empirical forgetting rate and log-likelihood values. Fig. 3 summarizes the results, which show that users, on average, achieve lower empirical forgetting rates whenever they follow MEMORIZE more closely.

Since the Leitner system (9), there have been a wealth of spaced repetition algorithms (7, 8, 10, 11, 13). However, there has been a paucity of work on designing adaptive data-driven spaced repetition algorithms with provable guarantees. In this work, we have introduced a principled modeling framework to design online spaced repetition algorithms with provable guarantees, which are specially designed to adapt to the learners’ performance, as monitored by modern spaced repetition software and online platforms. Our modeling framework represents spaced repetition using the framework of marked temporal point processes and SDEs with jumps and, exploiting this representation, it casts the design of spaced repetition algorithms as a stochastic optimal control problem of such jump SDEs. Since our framework is agnostic to the particular modeling choices, i.e., the memory model and the quadratic loss function, we believe it provides a powerful tool to find spaced repetition algorithms that are provably optimal under a given choice of memory model and loss.

There are many interesting directions for future work. For example, it would be interesting to perform large-scale interventional experiments to assess the performance of our algorithm in comparison with existing spaced repetition algorithms deployed by, e.g., Duolingo. Moreover, in our work, we consider a particular quadratic loss and soft constraints on the number of reviewing events; however, it would be useful to derive optimal reviewing intensities for other losses capturing particular learning goals and hard constraints on the number of events. We assumed that, by reviewing an item, one can influence only its recall probability and forgetting rate. However, items may be dependent and, by reviewing an item, one can influence the recall probabilities and forgetting rates of several items. The dataset we used spans only 2 wk and that places a limitation on the range of time intervals between reviews and retention intervals we can study. It would be very interesting to evaluate our framework in datasets spanning longer periods of time. Finally, we believe that the mathematical techniques underpinning our algorithm, i.e., stochastic optimal control of SDEs with jumps, have the potential to drive the design of control algorithms in a wide range of applications.

## Materials and Methods

### Evaluation Procedure.

To evaluate performance of our proposed algorithm, we rely on the following evaluation procedure. For each (user, item) reviewing sequence, we first perform a likelihood-based comparison and determine how closely it follows a specific reviewing schedule (be it MEMORIZE, uniform, or threshold) during the first n−1 reviews, the training reviews, where n is the number of reviews in the reviewing sequence. Second, we compute a quality metric, empirical forgetting rate $\hat{n}\left(t_{n}\right)$, using the last review, the nth review or test review, and the retention interval $t_{n}-t_{n-1}$. Third, for each reviewing sequence, we record the value of the quality metric, the training period (i.e., $T=t_{n-1}-t_{1}$), and the likelihood under each reviewing schedule. Finally, we control for the training period and the number of reviewing events and create the treatment and control groups by picking the top 25% of pairs in terms of likelihood for each method, where we skip any sequence lying in the top 25% for more than one method. Refer to *SI Appendix*, section 13 for an additional analysis showing that our evaluation procedure satisfies the random assignment assumption for the item difficulties between treatment and control groups (31).

In the above procedure, to do the likelihood-based comparison, we first estimate the parameters α and β and the initial forgetting rate $n_{i}\left(0\right)$ using half-life regression on the Duolingo dataset. Here, note that we fit a single set of parameters α and β for all items and a different initial forgetting rate $n_{i}\left(0\right)$ per item i, and we use the power-law forgetting curve model due to its better performance (in terms of MAE) in our experiments (refer to *SI Appendix*, section 8 for more details). Then, for each user, we use maximum-likelihood estimation to fit the parameter q in MEMORIZE and the parameter μ in the uniform reviewing schedule. For the threshold-based schedule, we fit one set of parameters c and ζ for each sequence of review events, using maximum-likelihood estimation for the parameter c and grid search for the parameter ζ, and we fit one parameter $m^{th}$ for each user using grid search. Finally, we compute the likelihood of the times of the n−1 reviewing events for each (user, item) pair under the intensity given by MEMORIZE, i.e., $u\left(t\right)=q^{-1/2}\left(1-m\left(t\right)\right)$; the intensity given by the uniform schedule, i.e., u(t)=μ; and the intensity given by the threshold-based schedule, i.e., u(t)=c⁡exp((t−s)/ζ). The likelihood $LL\left(\left\{t_{i}\right\}\right)$ of a set of reviewing events $\left\{t_{i}\right\}$ given an intensity function u(t) can be computed as follows (16):

$$LL\left(\left\{t_{i}\right\}\right)=\underset{i}{\sum }\log u\left(t_{i}\right)-\int_{0}^{T}u\left(t\right)\;dt.$$

More details on the empirical distribution of likelihood values under each reviewing schedule are provided in *SI Appendix*, section 10.

### Quality Metric: Empirical Forgetting Rate.

For each (user, item), the empirical forgetting rate is an empirical estimate of the forgetting rate by the time $t_{n}$ of the last reviewing event; i.e.,

$$\hat{n}=-\log\left(\hat{m}\left(t_{n}\right)\right)/\left(t_{n}-t_{n-1}\right),$$

where $\hat{m}\left(t_{n}\right)$ is the empirical recall probability, which consists of the fraction of correct recalls of sentences containing word (item) i in the session at time $t_{n}$. Note that this empirical estimate does not depend on the particular choice of memory model and, given a sequence of reviews, the lower the empirical forgetting rate is, the more effective the reviewing schedule.

Moreover, for a more fair comparison across items, we normalize each empirical forgetting rate using the average empirical initial forgetting rate of the corresponding item at the beginning of the observation window ${\hat{n}}_{0}$; i.e., for an item i,

$${\hat{n}}_{0}=\frac{1}{\left|D_{i}\right|}\underset{\left(u,i\right)\in D_{i}}{\sum }{\hat{n}}_{0,\left(u,i\right)},$$

where $D_{i}\subseteq D$ is the subset of (user, item) pairs in which item i was reviewed. Furthermore, ${\hat{n}}_{0,\left(u,i\right)}=-\log\left(\hat{m}\left(t_{\left(u,i\right),1}\right)\right)/\left(t_{\left(u,i\right),1}-t_{\left(u,i\right),0}\right)$, where $t_{\left(u,i\right),k}$ is the kth review in the reviewing sequence associated to the (u,i) pair. However, our results are not sensitive to this normalization step, as shown in *SI Appendix*, section 14.

## Supplementary Material

Supplementary File

## Footnotes

^†^To derive Eq. **3**, we assume that the recall probability $m_{i}\left(t\right)$ is set to 1 every time item i is reviewed. Here, one may also account for item difficulty by considering that, for more difficult items, the recall probability is set to $o_{i}\in \left[0,1\right)$ every time item i is reviewed.
