# Supplementary material for: Enhancing human learning via spaced repetition optimization
Source: Proc Natl Acad Sci U S A. 2019 Jan 22;116(10):3988–93. doi: 10.1073/pnas.1815156116 (PMC6410796; doi:10.1073/pnas.1815156116)
Supplement: Supplementary File [file pnas.1815156116.sapp.pdf]

PNAS
www.pnas.org

Supplementary Information for

Supplementary Information for
Enhancing Human Learning via Spaced Repetition Optimization

Behzad Tabibian, Utkarsh Upadhyay, Abir De, Ali Zarezade, Bernhard Schölkopf, Manuel Gomez-Rodriguez

Behzad Tabibian.
E-mail: me@btabibian.com

This PDF file includes:

Supplementary text
Figs. S1 to S7
Tables S1 to S3
References for SI reference citations

www.pnas.org/cgi/doi/10.1073/pnas.1815156116

Supporting Information Text

1. Proof of Proposition 1

According to Eq. 1, the recall probability $m(t)$ depends on the forgetting rate, $n(t)$, and the time elapsed since the last review, $D(t) := t - t_r$. Moreover, we can readily write the differential of $D(t)$ as $dD(t) = dt - D(t)dN(t)$.

We define the vector $\boldsymbol{X}(t) = [n(t), D(t)]^T$. Then, we use Eq. 2 and Itö's calculus (1) to compute its differential:

$$\begin{aligned} d\boldsymbol{X}(t) &\overset{\text{dt}}{\underset{zol}{=}} \boldsymbol{f}(\boldsymbol{X}(t), t)dt + \boldsymbol{h}(\boldsymbol{X}(t), t)dN(t) \\ \boldsymbol{f}(\boldsymbol{X}(t), t) &= \left[\begin{smallmatrix} 0 \\ 1 \end{smallmatrix}\right] \\ \boldsymbol{h}(\boldsymbol{X}(t), t) &= \left[\begin{smallmatrix} -\alpha n(t) r(t) + \beta n(t)(1-r(t)) \\ -D(t) \end{smallmatrix}\right] \end{aligned}$$

Finally, using again Itö's calculus and the above differential, we can compute the differential of the recall probability $m(t) = e^{-n(t)D(t)} := F(\boldsymbol{X}(t))$ as follows:

$$\begin{aligned} dF(\boldsymbol{X}(t)) &= F(\boldsymbol{X}(t+dt)) - F(\boldsymbol{X}(t)) \\ &= F(\boldsymbol{X}(t) + d\boldsymbol{X}(t)) - F(\boldsymbol{X}(t)) \\ &\overset{\text{dt}}{\underset{zol}{=}} (f^T F_{\boldsymbol{X}}(\boldsymbol{X}(t)))dt + F(\boldsymbol{X}(t) + \boldsymbol{h}(\boldsymbol{X}(t), t)dN(t)) - F(\boldsymbol{X}(t)) \\ &\overset{\text{dt}}{\underset{zol}{=}} (f^T F_{\boldsymbol{X}}(\boldsymbol{X}(t)))dt + (F(\boldsymbol{X}(t) + h(\boldsymbol{X}(t), t)) - F(\boldsymbol{X}(t)))dN(t) \\ &= (e^{-(D(t)-D(t))n(t)(1+\alpha r_i(t) - \beta(1-r_i(t)))} - e^{-D(t)n(t)})dN(t) - n(t)e^{-D(t)n(t)}dt \\ &= -n(t)e^{-D(t)n(t)}dt + (1 - e^{-D(t)n(t)})dN(t) \\ &= -n(t)F(X(t))dt + (1 - F(X(t)))dN(t) \\ &= -n(t)m(t)dt + (1 - m(t))dN(t). \end{aligned}$$

2. Lemma 1

Lemma 1 *Let $x(t)$ and $y(t)$ be two jump-diffusion processes defined by the following jump SDEs:*

$$\begin{aligned} dx(t) &= f(x(t), y(t), t)dt + g(x(t), y(t), t)z(t)dN(t) + h(x(t), y(t), t)(1 - z(t))dN(t) \\ dy(t) &= p(x(t), y(t), t)dt + q(x(t), y(t), t)dN(t) \end{aligned}$$

where $N(t)$ is a jump process and $z(t) \in \{0, 1\}$. If function $F(x(t), y(t), t)$ is once continuously differentiable in $x(t)$, $y(t)$ and t, then,

$$dF(x, y, t) = (F_t + fF_x + pF_y)(x, y, t)dt + [F(x + g, y + q, t)z(t) + F(x + h, y + q, t)(1 - z(t)) - F(x, y, t)]dN(t),$$

where for notational simplicity we dropped the arguments of the functions f, g, h, p, q and argument of state variables.

Proof According to the definition of differential,

$$\begin{aligned} dF := dF(x(t), y(t), t) &= F(x(t+dt), y(t+dt), t+dt) - F(x(t), y(t), t) \\ &= F(x(t) + dx(t), y(t) + dy(t), t + dt) - F(x(t), y(t), t). \end{aligned}$$

Then, using Itö's calculus, we can write

$$\begin{aligned} dF \overset{\text{dt}}{\underset{zol}{=}} & F(x + fdt + g, y + pdt + q, t + dt)dN(t)z + F(x + fdt + h, y + pdt + q, t + dt)dN(t)(1 - z) \\ & + F(x + fdt, y + pdt, t + dt)(1 - dN(t)) - F(x, y, t) \end{aligned} \qquad [1]$$

where for notational simplicity we drop arguments of all functions except F and dN. Then, we expand the first three terms:

$$\begin{aligned} F(x + fdt + g, y + pdt + q, t + dt) &= F(x + g, y + q, t) + F_x(x + g, y + q, t)fdt \\ &\quad + F_y(x + g, y + q, t)pdt + F_t(x + g, y + q, t)dt \\ F(x + fdt + h, y + pdt + q, t + dt) &= F(x + h, y + q, t) + F_x(x + h, y + q, t)fdt \\ &\quad + F_y(x + h, y + q, t)pdt + F_t(x + h, y + q, t)dt \\ F(x + fdt, y + pdt, t + dt) &= F(x, y, t) + F_x(x, y, t)fdt + F_y(x, y, t)pdt \\ &\quad + F_t(x, y, t)dt \end{aligned}$$

using that the bilinear differential form $dt\, dN(t) = 0$. Finally, by substituting the above three equations into Eq. 1, we conclude that:

$$\begin{aligned} dF(x(t), y(t), t) = (F_t + fF_x + pF_y)(x(t), y(t), t)dt + \Big[& F(x + g, y + q, t)z(t) + F(x + h, y + q, t)(1 - z(t)) \\ & - F(x, y, t)\Big]dN(t), \end{aligned}$$

■

Specifically, consider $x(t) = n(t)$, $y(t) = m(t)$, $z(t) = r(t)$ and $J = F$ from equation 6 in the above equation, then,

$$dJ(m,n,t) = J_t(m,n,t) - nmJ_m(m,n,t) + [J(1,(1-\alpha)n,t)r(t) + J(1,(1+\beta)n,t)(1-r) \\ - J(m,n,t)]dN(t).$$

3. Lemma 2

Lemma 2 *Consider the following family of losses with parameter $d > 0$,*

$$\begin{aligned} \ell_d(m(t),n(t),u(t)) &= h_d(m(t),n(t)) + g_d^2(m(t),n(t)) + \frac{1}{2}qu(t)^2, \\ g_d(m(t),n(t)) &= 2^{-1/2}\left[c_2\frac{\log(d)}{-m(t)^2+2m(t)-d} - c_2\frac{\log(d)}{1-d} + c_1 m(t)\log\left(\frac{1+\beta}{1-\alpha}\right) - c_1\log(1+\beta)\right]_+, \\ h_d(m(t),n(t)) &= -\sqrt{q}m(t)n(t)c_2\frac{(-2m(t)+2)\log(d)}{(-m(t)^2+2m(t)-d)^2}. \end{aligned} \quad [2]$$

where $c_1, c_2 \in \mathbb{R}$ are arbitrary constants. Then, the cost-to-go $J_d(m(t),n(t),t)$ that satisfies the HJB equation, defined by Eq. 7, is given by:

$$J_d(m(t),n(t),t) = \sqrt{q}\left(c_1\log(n(t)) + c_2\frac{\log(d)}{-m(t)^2+2m(t)-d}\right) \quad [3]$$

and the optimal intensity is given by:

$$u_d^*(t) = q^{-1/2}[c_2\frac{\log(d)}{-m(t)^2+2m(t)-d} - c_2\frac{\log(d)}{1-d} + c_1 m(t)\log\left(\frac{1+\beta}{1-\alpha}\right) - c_1\log(1+\beta)]_+.$$

Proof *Consider the family of losses defined by Eq. 2 and the functional form for the cost-to-go defined by Eq. 3. Then, for any parameter value $d > 0$, the optimal intensity $u_d^*(t)$ is given by*

$$\begin{aligned} u_d^*(t) &= q^{-1}\left[J_d(m(t),n(t),t) - J_d(1,(1-\alpha)n(t),t)m(t) - J_d(1,(1+\beta)n(t),t)(1-m(t))\right]_+ \\ &= q^{-1/2}\left[c_2\frac{\log(d)}{-m^2+2m-d} - c_2\frac{\log(d)}{1-d} + c_1 m(t)\log\left(\frac{1+\beta}{1-\alpha}\right) - c_1\log(1+\beta)\right]_+, \end{aligned}$$

and the HJB equation is satisfied:

$$\begin{aligned} &\frac{\partial J_d(m,n,t)}{\partial t} - mn\frac{\partial J_d(m,n,t)}{\partial m} + h_d(m,n) + g_d^2(m,n) - \frac{1}{2}q^{-1}\big(J_d(m,n,t) \\ &- J_d(1,(1-\alpha)n,t)m - J_d(1,(1+\beta)n,t)(1-m)\big)_+^2 \\ &= \sqrt{q}mnc_2\frac{(-2m+2)\log(d)}{(-m^2+2m-d)^2} + h_d(m,n) + g_d^2(m,n) - \frac{1}{2}\Bigg[c_1\log(n) + c_2\frac{\log(d)}{-m^2+2m-d} \\ &\quad -m\left(c_1\log(n(1-\alpha)) + c_2\frac{\log(d)}{1-d}\right) - (1-m)\left(c_1\log(n(1+\beta)) + c_2\frac{\log(d)}{1-d}\right)\Bigg]_+^2 \\ &= \sqrt{q}mnc_2\frac{(-2m+2)\log(d)}{(-m^2+2m-d)^2} \underbrace{-\sqrt{q}mnc_2\frac{(-2m+2)\log(d)}{(-m^2+2m-d)^2}}_{h_d(m,n)} \\ &\quad -\frac{1}{2}\left[c_2\frac{\log(d)}{-m^2+2m-d} - c_2\frac{\log(d)}{1-d} + c_1 m\log(\frac{1+\beta}{1-\alpha}) - c_1\log(1+\beta)\right]_+^2 \\ &\quad +\frac{1}{2}\left[c_2\frac{\log(d)}{-m^2+2m-d} - c_2\frac{\log(d)}{1-d} + c_1 m\log(\frac{1+\beta}{1-\alpha}) - c_1\log(1+\beta)\right]_+^2 \end{aligned}$$

where for notational simplicity $m = m(t)$, $n = n(t)$ and $u = u(t)$. ■

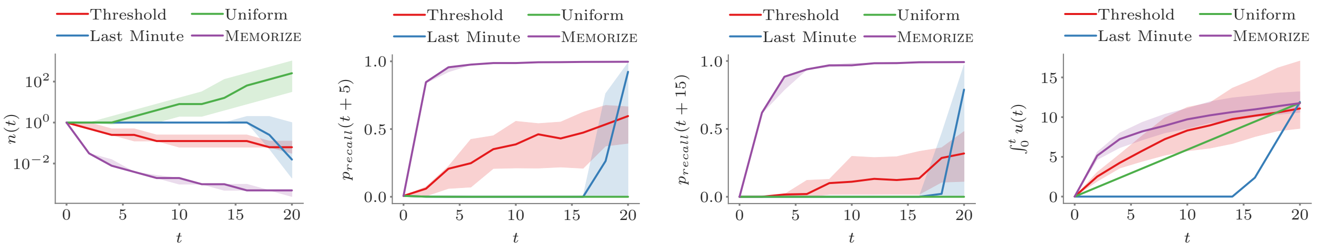

(a) Forgetting rate **(b)** Short-term recall prob. **(c)** Long-term recall prob. **(d)** Reviewing intensity

Fig. S1. Performance of MEMORIZE in comparison with several baselines. The solid lines are median values and the shadowed regions are 30% confidence intervals. Short-term recall probability corresponds to $m(t+5)$ and long-term recall probability to $m(t+15)$. In all cases, we use $\alpha = 0.5, \beta = 1, n(0) = 1$ and $t_f - t_0 = 20$. Moreover, we set $q = 3 \cdot 10^{-4}$ for MEMORIZE, $\mu = 0.6$ for the uniform reviewing schedule, $t_{lm} = 5$ and $\mu = 2.38$ for the last minute reviewing schedule, and $m^{th} = 0.7$ and $c = \zeta = 5$ for the threshold based reviewing schedule. Under these parameter values, the total number of reviewing events for all algorithms are equal (with a tolerance of 5%).

4. Proof of Theorem 3

Consider the family of losses defined by Eq. 2 in Lemma 2 whose optimal intensity is given by:

$$u_d^*(t) = q^{-1/2}\left[c_2 \frac{\log(d)}{-m^2+2m-d} - c_2 \frac{\log(d)}{1-d} + c_1 m(t) \log\left(\frac{1+\beta}{1-\alpha}\right) - c_1 \log(1+\beta)\right]_+ .$$

Now, set the constants $c_1, c_2 \in \mathbb{R}$ to the following values:

$$c_1 = \frac{-1}{\log\left(\frac{1+\beta}{1-\alpha}\right)} \quad c_2 = \frac{-\log(1-\alpha)}{\log\left(\frac{1+\beta}{1-\alpha}\right)}.$$

Since the HJB equation is satisfied for any value of $d > 0$, we can recover the quadratic loss $l(m, n, u)$ and derive its corresponding optimal intensity $u^*(t)$ using point wise convergence:

$$l(m(t), n(t), u(t)) = \lim_{d \to 1} l_d(m(t), n(t), u(t)) = \frac{1}{2}(1-m(t))^2 + \frac{1}{2} q u^2(t),$$
$$u^*(t) = \lim_{d \to 1} u_d^*(t) = q^{-1/2}(1-m(t)),$$

where we used that $\lim_{d\to 1} \frac{\log(d)}{1-d} = -1$ (L'Hospital's rule).

This concludes the proof.

5. Synthetic Experiments

In this section, our goal is analyzing the performance of MEMORIZE under a controlled setting using metrics and baselines that we cannot compute in the real data we have access to. We evaluate the performance of MEMORIZE using two quality metrics: recall probability $m(t+\tau)$ at a given time in the future $t+\tau$ and forgetting rate $n(t)$. Here, by considering high (low) values of τ, we can assess long-term (short-term) retention. Moreover, we compare the performance of our algorithm with three baselines: (i) a *uniform* reviewing schedule, which sends item(s) for review at a constant rate μ; (ii) a *threshold* based reviewing schedule, which increases the reviewing intensity of an item by $c\exp((t-s)/\zeta)$ at time s, when its recall probability reaches a threshold m^{th}; and, (iii) a *last minute* reviewing schedule, which only sends item(s) for review during a period $[t_{lm}, t_f]$, at a constant rate μ therein.* Unless otherwise stated, we set the parameters of the baselines and our algorithm such that the total number of reviewing events during $(t_0, t_f]$ are equal.

First, we run 100 independent simulations and compute the above quality metrics over time. Figure S1 summarizes the results, which show that our model: (i) consistently outperforms all the baselines in terms of both quality metrics; (ii) is more robust across runs both in terms of quality metrics and reviewing schedule; and (iii) reduces the reviewing intensity as times goes by and the recall probability improves, as one could have expected.

Second, we experiment with different values for the parameter q, which controls the learning effort required by MEMORIZE—the lower its value, the higher the number of reviewing events. Intuitively, one may also expect the learning effort to influence how quickly a learner memorizes a given item—the lower its value, the quicker a learner will memorize it. Figure S2a confirms this intuition by showing the average forgetting rate $n(t)$ and number of reviewing events $N(t)$ at several times t for different q values.

Finally, we experiment with different values for the parameters α and β, which capture the aptitude of a learner and the difficulty of the item to be learned—the higher (lower) the value of α (β), the quicker a learner will memorize the item. In Figure S2b, we evaluate quantitatively this effect by means of the average time the learner takes to reach a forgetting rate of $n(t) = \frac{1}{2}n(0)$ using MEMORIZE for different parameter values.

*The last minute reviewing schedule is only introduced here and was not used in empirical evaluations since in Duolingo there is no terminal time t_f which users target. Additionally, in many (user, item) pairs, the first review takes place close to $t = 0$ and thus the last minute baseline is equivalent to the uniform reviewing schedule.

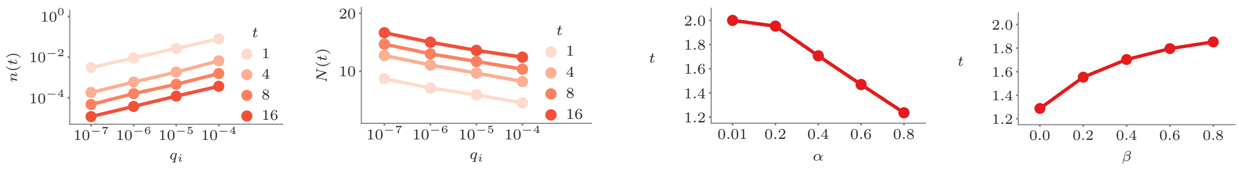

(a) Learning effort

(b) Item difficulty and aptitude of the learner

Fig. S2. Learning effort, aptitude of the learner and item difficulty. Panel (a) shows the average forgetting rate $n(t)$ and number of reviewing events $N(t)$ for different values of the parameter q, which controls the learning effort. Panel (b) shows the average time the learner takes to reach a forgetting rate $n(t) = \frac{1}{2}n(0)$ for different values of the parameters α and β, which capture the aptitude of the learner and the item difficulty. In Panel (a), we use $\alpha = 0.5$, $\beta = 1$, $n(0) = 1$ and $t_f - t_0 = 20$. In Panel (b), we use $n(0) = 20$ and $q = 0.02$. In both panels, error bars are too small to be seen.

6. Our Modeling Framework Using the Power-Law Forgetting Curve Model

Under the power-law forgetting curve model, the probability of recalling an item i at time t is given by (2):

$$m_i(t) := \mathbb{P}(r_i(t)) = (1 + \omega(t - t_r))^{-n_i(t)}, \quad [4]$$

where t_r is the time of the last review, $n_i(t) \in RR^+$ is the forgetting rate and ω is a time scale parameter.

Similarly as in Proposition 1 for the exponential forgetting curve model, we can express the dynamics of the recall probability $m_i(t)$ by means of a SDE with jumps:

$$dm_i(t) = -\frac{n_i(t)m_i(t)\omega dt}{(1 + \omega D_i(t))} + (1 - m_i(t))dN_i(t) \quad [5]$$

where $D_i(t) := t - t_r$ and thus the differential of $D_i(t)$ is readily given by $dD_i(t) = dt - D_i(t)dN_i(t)$.

Next, similarly as in the case of the exponential forgetting curve model in the main paper, we consider a single item with $n_i(t) = n(t)$, $m_i(t) = m(t)$, $D_i(t) = D(t)$ and $r_i(t) = r(t)$, and adapt Lemma 1 to the power-law forgetting curve model as follows:

Lemma 3 *Let $x(t)$ and $y(t)$, $k(t)$ be three jump-diffusion processes defined by the following jump SDEs:*

$$\begin{aligned} dx(t) =& f(x(t), y(t), t)dt + g(x(t), y(t), t)z(t)dN(t) + h(x(t), y(t), t)(1 - z(t))dN(t) \\ dy(t) =& p(x(t), y(t), t)dt + q(x(t), y(t), t)dN(t) \\ dk(t) =& s(x(t), y(t), k(t), t)dt + v(x(t), y(t), k(t), t)dN(t) \end{aligned}$$

where $N(t)$ is a jump process and $z(t) \in \{0, 1\}$. If function $F(x(t), y(t), k(t), t)$ is once continuously differentiable in $x(t)$, $y(t)$, $z(t)$ and t, then,

$$\begin{aligned} dF(x, y, k, t) = (F_t + fF_x + pF_y + sF_s)(x, y, k, t)dt + [F(x + g, y + q, k + v, t)z(t) \\ + F(x + h, y + q, k + v, t)(1 - z(t)) - F(x, y, t)]dN(t), \end{aligned}$$

where for notational simplicity we dropped the arguments of the functions f, g, h, p, q, s, v and argument of state variables.

Then, if we consider $x(t) = n(t)$, $y(t) = m(t)$, $k(t) = D(t)$, $z(t) = r(t)$ and $J = F$ in the above Lemma, the differential of the optimal cost-to-go is readily given by

$$\begin{aligned} dJ(m, n, t) = J_t(m, n, t) - \frac{\omega nm}{1 + \omega D} J_m(m, n, D, t) + J_D(m, n, D, t) + [J(1, (1 - \alpha)n, 0, t)r(t) \\ + J(1, (1 + \omega)n, 0, t)(1 - r) - J(m, n, D, t)]dN(t). \end{aligned}$$

Moreover, under the same loss function $\ell(m(t), n(t), u(t))$ as in Eq. 8, it is easy to show that the optimal cost-to-go J needs to satisfy the following nonlinear partial differential equation:

$$\begin{aligned} 0 = J_t(m(t), n(t), t) - \frac{\omega n(t)m(t)}{1 + \omega D} J_m(m(t), n(t), t) + \quad J_D(m(t), n(t), t) + \frac{1}{2}(1 - m(t))^2 \\ - \frac{1}{2}q^{-1}\big(J(m(t), n(t), t) - J(1, (1 - \alpha)n(t), t)m(t) - J(1, (1 + \beta)n(t), t)(1 - m(t))\big)_+^2. \quad [6] \end{aligned}$$

Then, we can adapt Lemma 2 to derive the optimal scheduling policy for a single item under the power-law forgetting curve model:

Lemma 4 *Consider the following family of losses with parameter $d > 0$,*

$$\begin{aligned} \ell_d(m(t), n(t), D(t), u(t)) &= h_d(m(t), n(t), D(t)) + g_d^2(m(t), n(t)) + \frac{1}{2} q u(t)^2, \\ g_d(m(t), n(t)) &= 2^{-1/2} \left[c_2 \frac{\log(d)}{-m(t)^2 + 2m(t) - d} - c_2 \frac{\log(d)}{1-d} + c_1 m(t) \log\left(\frac{1+\beta}{1-\alpha}\right) - c_1 \log(1+\beta) \right]_+, \\ h_d(m(t), n(t)) &= -\sqrt{q} \frac{\omega n(t) m(t)}{1 + \omega D(t)} c_2 \frac{(-2m(t)+2)\log(d)}{(-m(t)^2 + 2m(t) - d)^2}. \end{aligned} \quad [7]$$

where $c_1, c_2 \in \mathbb{R}$ are arbitrary constants. Then, the cost-to-go $J_d(m(t), n(t), t)$ that satisfies the HJB equation, defined by Eq. 6, is given by:

$$J_d(m(t), n(t), D(t), t) = \sqrt{q} \left(c_1 \log(n(t)) + c_2 \frac{\log(d)}{-m(t)^2 + 2m(t) - d} \right) \quad [8]$$

which is independent of $D(t)$, and the optimal intensity is given by:

$$u_d^*(t) = q^{-1/2} [c_2 \frac{\log(d)}{-m(t)^2 + 2m(t) - d} - c_2 \frac{\log(d)}{1-d} + c_1 m(t) \log\left(\frac{1+\beta}{1-\alpha}\right) - c_1 \log(1+\beta)]_+.$$

Proof *Consider the family of losses defined by Eq. 7 and the functional form for the cost-to-go defined by Eq. 8. Then, for any parameter value $d > 0$, the optimal intensity $u_d^*(t)$ is given by*

$$\begin{aligned} u_d^*(t) &= q^{-1} \left[J_d(m(t), n(t), t) - J_d(1, (1-\alpha)n(t), t) m(t) - J_d(1, (1+\beta)n(t), t)(1 - m(t)) \right]_+ \\ &= q^{-1/2} \left[c_2 \frac{\log(d)}{-m^2 + 2m - d} - c_2 \frac{\log(d)}{1-d} + c_1 m(t) \log\left(\frac{1+\beta}{1-\alpha}\right) - c_1 \log(1+\beta) \right]_+, \end{aligned}$$

and the HJB equation is satisfied:

$$\begin{aligned} &\frac{\partial J_d(m,n,t)}{\partial t} - \frac{\omega n m}{1+\omega D} \frac{\partial J_d(m,n,t)}{\partial m} + h_d(m,n) + g_d^2(m,n) - \frac{1}{2} q^{-1} \big(J_d(m,n,t) \\ &- J_d(1, (1-\alpha)n, t) m - J_d(1, (1+\beta)n, t)(1-m) \big)_+^2 \\ &= \sqrt{q} \frac{\beta c_2 n m}{1+\omega D} \frac{(-2m+2)\log(d)}{(-m^2+2m-d)^2} + h_d(m,n) + g_d^2(m,n) - \frac{1}{2} \Bigg[c_1 \log(n) + c_2 \frac{\log(d)}{-m^2+2m-d} \\ &\quad -m \left(c_1 \log(n(1-\alpha)) + c_2 \frac{\log(d)}{1-d} \right) - (1-m) \left(c_1 \log(n(1+\beta)) + c_2 \frac{\log(d)}{1-d} \right) \Bigg]_+^2 \\ &= \sqrt{q} \frac{\omega n m c_2}{1+\omega D(t)} \frac{(-2m+2)\log(d)}{(-m^2+2m-d)^2} \underbrace{- \sqrt{q} \frac{\omega n m c_2}{1+\omega D} \frac{(-2m+2)\log(d)}{(-m^2+2m-d)^2}}_{h_d(m,n)} \\ &\quad - \frac{1}{2} \big[c_2 \frac{\log(d)}{-m^2+2m-d} - c_2 \frac{\log(d)}{1-d} + c_1 m \log(\frac{1+\beta}{1-\alpha}) - c_1 \log(1+\beta) \big]_+^2 \\ &\quad + \frac{1}{2} \big[c_2 \frac{\log(d)}{-m^2+2m-d} - c_2 \frac{\log(d)}{1-d} + c_1 m \log(\frac{1+\beta}{1-\alpha}) - c_1 \log(1+\beta) \big]_+^2 \end{aligned}$$

where for notational simplicity $m = m(t)$, $n = n(t)$, $D = D(t)$ and $u = u(t)$. ■

Finally, reusing Theorem 3, the optimal reviewing intensity for a single item under the power-law forgetting curve model is given by

$$u^*(t) = \lim_{d \to 1} u_d^*(t) = q^{-1/2}(1 - m(t)).$$

It is then straightforward to derive the optimal reviewing intensity for a set of items, which adopts the same form as in Theorem 4.

	HLR Exponential	Our Model Exponential	Our Model Power-law
MAE↓	0.128	0.129	**0.105**
AUC↑	0.538	**0.542**	0.533
COR$_h$ ↑	**0.201**	0.165	0.123

Table S1. Predictive performance of the exponential and power-law forgetting curve models in comparison with the results reported by Settles et al. (4). The arrows indicate whether a higher value of the metric is better (↑) or a lower value (↓).

7. Our Modeling Framework Using the Multiscale Context Model

In this section, we will briefly describe the Multiscale Context Model (MCM) of memory (3) and sketch how derive the optimal reviewing schedule for this model.

MCM models memory as *activation* of M time-varying *context* units with the values $\{x_i(t)\}_{[M]}$. The units are *leaky* and lose their activation at different decay rates $\{\tau_i\}_{[M]}; \tau_{i+1} > \tau_i$, such that $\Delta x_i(t + \Delta t) = x_i(t) \exp\left(-\Delta t/\tau_i\right)$. The accumulated activity of the units, each scaled by $\{\gamma_i\}_{[M]}$, represents the total *trace strength* of the item at time t, *i.e.*, $s_M(t) = \frac{\sum_{i=0}^{M} \gamma_i x_i(t)}{\sum_{i=0}^{M} \gamma_i}$. The probability of recall of the item at time t is then calculated as $m_{MCM}(t) = \min\{1, s_M(t)\}$.

Each time a review happens, the activation of the context pools *jumps* by an amount which depends on how much activation at timescale i, *i.e.*, $x_i(t)$ had *contributed* to the recall. So if a review happens at time t_r, $\Delta x_i(t_r^+) = \varepsilon(1 - s_i(t_r^-))$, where $s_i(t)$ represents the strength of all activations which decay *faster* than the current trace, *i.e.*, $s_i(t) = \frac{\sum_{j=1}^{i} \gamma_j x_j(t)}{\sum_{j=1}^{i} \gamma_j}$. The value of ε is set to ε_α if the recall was successful and to ε_β if the recall was unsuccessful. For a more detailed description of MCM, we refer the readers to (3).

The dynamics of the context pools $\{x_i(t)\}_{[M]}$ can be converted to the corresponding SDEs readily as follows.

$$s_i(t) = \frac{\sum_{j=1}^{i} \gamma_j x_j(t)}{\sum_{j=1}^{i} \gamma_j}$$
$$dx_i(t) = \varepsilon_\alpha r(t)(1 - s_i(t))dN(t) + \varepsilon_\beta(1 - r(t))(1 - s_i(t))dN(t) - \frac{x_i(t)}{\tau_i}dt \qquad [9]$$
$$ds_M(t) = \frac{\sum_{j=1}^{M} \gamma_j dx_j(t)}{\sum_{j=1}^{M} \gamma_j}.$$

For modeling the probability of recall $m_{MCM}(t)$, we can use a differentiable approximation to the $\min\{1, s_M(t)\}$ function. For example, we can use hyperbolic-tan, and approximate $m_{MCM}(t)$ via $\tilde{m}_{MCM}(t)$:

$$\tilde{m}_{MCM}(t) = \tanh s_M(t)$$
$$\implies d\tilde{m}_{MCM}(t) = (1 - \tilde{m}^2_{MCM}(t))ds_M(t). \qquad [10]$$

One can contrast Eq. 9 and Eq. 10 with Eq. 2 and Eq. 3 (or Eq. 5) respectively to compare the derivations for the exponential forgetting curve model and the MCM.

Extension of Lemma 2 for Eq. 10 is straight-forward and the nonlinear partial differential equation corresponding to Eq. 7 (or Eq. 6) can be solved to arrive at the optimal scheduling for the MCM model. The resulting equation, however, does not readily admit to an analytical solution as was the case for the exponential and power-law forgetting curve models.

8. Predictive performance of the memory model

Before we evaluate the predictive performance of the exponential and power-law forgetting curve models, whose forgetting rates we estimated using a variant of Half-life regression (HLR) (4), we highlight the differences between the original HLR and the variant we used.

The original HLR and the variant we used differ in the way successful and unsuccessful recalls change the forgetting rate. In our work, the forgetting rate at time t depends on $n_{\checkmark}(t) = \int_0^t r(\tau)dN(\tau)$ and $n_{\times}(t) = \int_0^t (1 - r(\tau))dN(\tau)$. In contrast, in the original HLR, the forgetting rate at time t depends on $\sqrt{n_{\checkmark}(t) + 1}$ and $\sqrt{n_{\times}(t) + 1}$. The rationale behind our modeling choice is to be able to express the dynamics of the forgetting rate using a linear stochastic differential equation with jumps. Moreover, Settles *et al.* consider each session to contain multiple review events for each item. Hence, within a session, the $n_{\times}(t)$ and $n_{\checkmark}(t)$ may increase by more than one. In contrast, we consider each session to contain a single review event for each item because the reviews in each session take place in a very short time and it is likely that after the first review, the user will recall the item correctly during that session. Hence, we only increase one of $n_{\checkmark}(t)$ or $n_{\times}(t)$ by exactly 1 after each session and consider an item has been successfully recalled during a session if all reviews were successful, *i.e.*, $p_{recall} = 1$. Noticeably, ∼83% of the items were successfully recalled during a session.

K	Interval boundaries
3	[0, 20 minutes, 2.9 days, ∞]
4	[0, 9 minutes, 21.5 hours, 5.2 days, ∞]
5	[0, 6 minutes, 1.5 hours, 1.8 days, 7.3 days, ∞]

Table S2. The boundaries of intervals used to divide review times into bins based on K-quantiles of inter-review times in the Duolingo dataset.

(a) Comparing $\alpha^{(\text{row})}$ and $\alpha^{(\text{col})}$

	2	3	4	5
1	0.317	0.317	0.317	0.317
2		0.312	0.312	0.312
3			0.317	0.317
4				0.318

(b) Comparing $\beta^{(\text{row})}$ and $\beta^{(\text{col})}$

	2	3	4	5
1	0.165	0.172	0.165	0.165
2		0.318	0.302	0.302
3			0.318	0.318
4				0.302

Table S3. p-values obtained after using Welch's t-test for populations with different variance to reject the null hypothesis that the samples (*i.e.*, 400 samples of $\{\alpha^{(i)}\}$ and $\{\beta^{(i)}\}$ for $i \in [5]$) have the same mean value. In all cases, we find no evidence to reject the null hypothesis. The results for other values of K were qualitatively similar.

Table S1 summarizes our results on the Duolingo dataset in terms of mean absolute error (MAE), area under curve (AUC) and correlation (COR_h), which show that the performance of both the exponential and power-law forgetting curve models with forgetting rates estimated using the variant of HLR is comparable to the performance of the exponential forgetting curve model with forgetting rates estimated using the original HLR. In the above results, note that we fitted a single set of parameters α and β for all items and a different initial forgetting rate $n_i(0)$ per item i. One could think of estimating item specific (or even user specific) parameters α and β, however, we found that our dataset is not large enough to provide accurate estimates of such item specific parameters. More generally, there is always a trade-off between the complexity of the model and the size of the dataset used to fit the model parameters.

9. Constant vs time-varying α and β parameters

In previous studies (5, 6), it has been shown the retention rate follows an *inverted U-shape*, *i.e.*, *mass practice* does not improve the forgetting rate, and thus one could argue that our framework should consider time-varying parameters $\alpha_i(t)$ and $\beta_i(t)$. In this section, we show that, for the reviewing sequences in our Duolingo dataset, allowing for time-varying $\alpha_i(t)$ and $\beta_i(t)$ in our modeling framework does not lead to more accurate recall predictions. This was one of the reasons, in addition to tractability, for considering constant parameters α_i and β_i.

Formulation. In Eq. 2, we have considered α and β as constants, *i.e.*, they do not vary with the review interval $t - t_r$, where t_r is the time of last review. We have dropped the subscript i denoting the item for ease of exposition. We can make a zeroth-order approximation to time varying (α, β) by allowing them to be piecewise constant for K mutually exclusive and exhaustive review-time intervals $\left\{B^{(i)}\right\}_{[K]}$. We denote the value that α (β) takes in interval $B^{(i)}$ as $\alpha^{(i)}$ $(\beta^{(i)})$ and modify the forgetting rate update equation to

$$dn(t) = \underbrace{-\alpha^{(i)} n(t) r(t) dN(t) + \beta^{(i)} n(t)(1 - r(t)) dN(t)}_{i \text{ such that } t - t_r \in B^{(i)}}$$

If we find that $\exists \{i, j\} \subset [K]$ such that $\alpha^{(i)}$ $(\beta^{(i)})$ is *significantly* different from $\alpha^{(j)}$ $(\beta^{(j)})$, then we would conclude that α (β) vary with review-time.

We obtain repeated estimates of $\left\{\alpha^{(i)}\right\}_{[K]}$ and $\left\{\beta^{(i)}\right\}_{[K]}$ by fitting our model to datasets sampled with replacement from our Duolingo dataset, *i.e.*, via bootstrapping. The Welch's t-test is used to test if the difference in mean values of the parameters in different bins is significant.

Experimental setup. We set the bins boundaries by determining the K-quantiles of the review times in our dataset. Table S2 shows that the bin boundaries for different K are quite varied and adequately cover long time-scales as well as review intervals which are short enough to capture *massed practicing*. This method of binning also ensures that we have sufficient samples for accurate estimation ($\sim 5.2e6/K$) for all parameters. Then we use the variant of HLR described in Appendix 8 to fit the parameters in 400 different datasets using bootstrapping. The regularization parameters are determined via grid-search using a train/test dataset. We thus obtain 400 samples of $\left\{\alpha^{(i)}\right\}_{[K]}$ and $\left\{\beta^{(i)}\right\}_{[K]}$ for $K \in \{3, 4, 5\}$ and $i \in [K]$. Using Welch's t-test for distributions with varying variances, we observe that the mean values of the distributions of $\left\{\alpha^{(i)}\right\}_{[K]}$ $(\left\{\beta^{(i)}\right\}_{[K]})$ and $\left\{\alpha^{(j)}\right\}_{[K]}$ $(\left\{\beta^{(j)}\right\}_{[K]})$ are not significantly different for any $\{i, j\}$. As an example, the p-values obtained for $K = 5$ are shown in Table S3.

10. Likelihood distributions for different reviewing schedules

In this section, we compute the likelihood of each sequence of review events in our dataset under different reviewing schedules. Figure S3 summarizes the results by showing the empirical distribution of estimated likelihood values for MEMORIZE, threshold

and uniform schedules. Since Duolingo uses a near-optimal hand-tuned reviewing schedule, the peak of the distribution for MEMORIZE corresponds to the highest likelihood values, *i.e.*, there are many (user, item) pairs who follow MEMORIZE closely.

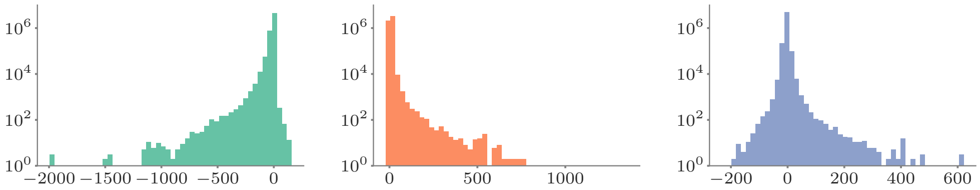

(a) MEMORIZE　　**(b)** Threshold　　**(c)** Uniform

Fig. S3. Empirical distribution of log-likelihood values for all (user, item) pairs under different reviewing schedules. Since Duolingo uses a near-optimal hand-tuned reviewing schedule, the peak of the distribution for MEMORIZE corresponds to the highest likelihood values, *i.e.*, there are many (user, item) pairs who follow MEMORIZE closely.

11. Leitner system: particular case of our modeling framework

In this section, we first describe the Leitner system and then show that it can be explicitly cast using our modeling framework with particular choices of α, β and $n_i(0)$.

Leitner system. More than 40 years ago, Sebastian Leitner introduced the Leitner System as a method used to memorize flash cards (7). Since then, several variants of the system have been introduced and some of them are still in active use. Next, for the sake of brevity, we describe one of these variants, which has been recently studied by Reddy *et al.* (8) and Settles *et al.* (4).

The learner maintains several *decks* of flashcards, labelled $j \in \mathbb{Z}$, each of which is reviewed at exponentially decreasing frequency $\lambda_j = \lambda_0 c^j$, for some constants c and λ_0. Whenever a card i from deck j is reviewed, it is moved to deck $j+1$ if it is recalled correctly (*i.e.*, if $r_i(t) = 1$), or else (if $r_i(t) = 0$) it is moved to deck $j-1$, as shown in Figure S4. The intuition behind the Leitner system is that cards which belong to a deck with a large index j have been learned (or were easy to learn), *i.e.*, they have a low forgetting rate, and cards which are in lower decks have not been learned yet (or were difficult to learn), *i.e.*, they have a high forgetting rate. Then, the learning strategy of the learner is to select flashcards at random from any deck as long as the reviewing rate for flashcards in each deck j remains λ_j, *i.e.*, the expected number of flashcards selected for review from deck j in any time interval Δt is $\lambda_j \times \Delta t$.

Modeling the Leitner system. For ease of exposition, we assume that the number of decks is unbounded both from above and below[†], *i.e.*, there are always decks with higher (or lower) rate of review than the current deck. Under this assumption, we can faithfully represent the Leitner system under our modeling framework as follows.

First, we assign a fixed forgetting rate $\tilde{n}_j$ to all flashcards in deck j, *i.e.*, if at time t, a flashcard i is in deck j then $n_i(t) = \tilde{n}_j$ and, at the beginning, all flashcards are placed in the first deck, *i.e.*, $\forall i. \, n_i(0) = \tilde{n}_0$. Then, every time a card i moves from deck j to $j+1$, we change its forgetting rate $n_i(t)$ by a factor of $(1-\alpha) = \frac{\tilde{n}_{j+1}}{\tilde{n}_j}$ and, similarly, every time it moves from j to $j-1$, we change it by a factor of $(1+\beta) = \frac{\tilde{n}_{j-1}}{\tilde{n}_j}$. Finally, we set $\lambda_j = \tilde{n}_j$, *i.e.*, the reviewing intensity of a card is proportional to the rate of forgetting associated to its deck, where the constant of proportionality has been absorbed into $\tilde{n}_j$. Now we can uncover α and β by solving the equations $(1-\alpha) = \frac{\tilde{n}_{j+1}}{\tilde{n}_j} = c$ and $(1+\beta) = \frac{\tilde{n}_{j-1}}{\tilde{n}_j} = \frac{1}{c}$. It is easy to see that, with minor modifications, our framework can be also used to represent many other variants of the Leitner system with, *e.g.*, bounded number of decks.

12. Additional details on the Duolingo dataset

Learners on Duolingo have a source language (which they already know) and a target language (which they wish to learn). Upon log-in, they are greeted with a screen to select the skill they wish to train/learn, shown in Figure S5a. As soon as they select a skill, a session begins (see Figure S5b). In each session, the learner is asked to translate ∼10 *phrases* from the source language to the target language or vice-versa. A typical session may last for ∼2–5 minutes but if it is interrupted in the middle due to any reason (loss of connectivity, student logging-out, *etc.*), the session (and its associated data) is discarded. Figures S5c and S5d show a correct translation and an incorrect translation, respectively, for one such phrases for the language pair (English, French). For each word that appears at least once in a session, our dataset contains identity of the learner, a timestamp (in UTC) indicating when the session started, the total number of times the word appears, and how many times the learner correctly/incorrectly translated the phrases containing that word. We consider a session to be a single point in time (localized at the start of the session), when the student practiced all the words appearing in it. A learner may do several sessions in a single sitting: the sessions in our dataset are separated by a median of ∼7 minutes, *including* the time the learner spent in the session.

[†] In a real deployment of the system, the bounds on the number of decks will be self imposed, *e.g.*, flashcards which move to a high enough deck will effectively never be studied, *i.e.*, they will be *retired*.

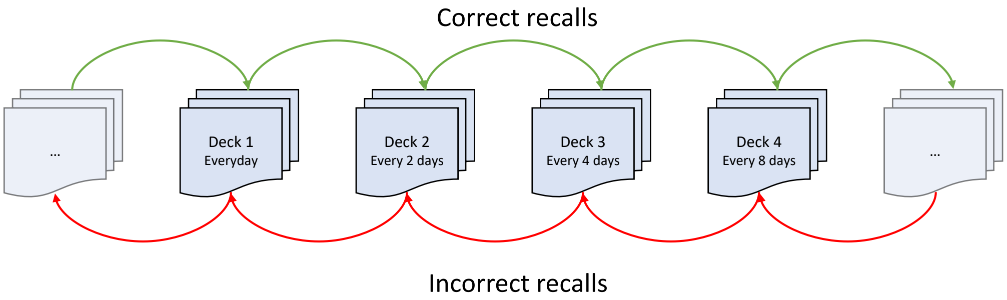

Fig. S4. The Leitner system. The learner picks flashcards for review from several decks and flashcards are moved from one deck to another based on the recall outcome after a review. The higher the index of the deck, the lower the rate at which cards are picked for review from that deck, *e.g.*, cards in deck 1 may be reviewed once per day, cards in deck 2 once every two days, and so on.

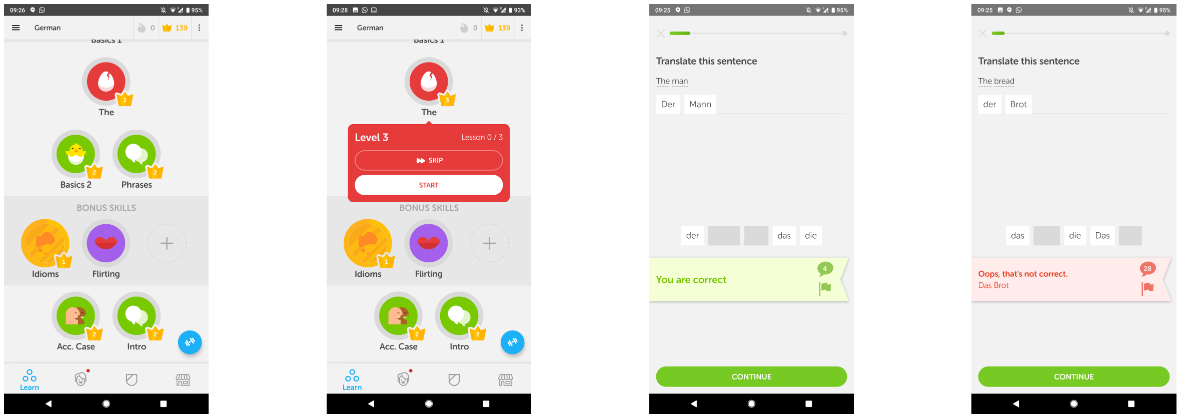

(a) Skill selection **(b)** Session start **(c)** Correct response **(d)** Incorrect response

Fig. S5. The Duolingo learning app.

As discussed in Section 8, our experimental design differs from that of Settles *et al.* (4) in some respects: we consider a word i to be recalled correctly at time t, *i.e.*, $r_i(t) = 1$, if the student answered *all* the questions containing that word correctly. Otherwise, we assume that the word was not recalled correctly.

13. Random assignment assumption on the item difficulties

To rule out that the competitive advantage that MEMORIZE offers with respect to the uniform and the threshold based baselines is a consequence of selection bias due to the item difficulty, we compute the empirical distributions of item difficulties for the treatment (MEMORIZE) and control (uniform and threshold) groups and check whether the allocation of items across the treatment and control groups resemble random assignment.

Figure S6 summarizes the results for reviewing sequences with a training period $T = 5 \pm 0.5$ days, which show a striking similarity between distributions across groups. In fact, the treatment group is *indistinguishable* from both the control groups in terms of item difficulties because their values are within a standardize mean difference (SMD)[‡] of 0.25 standard deviations (9). Similar results are obtained for sequences with a training period $T = 3 \pm 0.3$ and with $T = 7 \pm 0.7$.

Finally, we would like to acknowledge that there may be other covariates that influence the performance of a learner such as, *e.g.*, time of the day, amount of stress, amount of sleep or degree of concentration. Unfortunately, we did not have access to measurement about them.

14. Empirical forgetting rate without normalization

In Figure 2, for a more fair comparison across items, we normalized each empirical forgetting rate using the average empirical initial forgetting rate of the corresponding item at the beginning of the observation window $\hat{n}_0$. In this section, we demonstrate that the competitive advantage of our algorithm is not sensitive to this normalization step.

More specifically, we re-run our analysis using unnormalized values for the empirical forgetting rates. Figure S7 summarizes the results, which still show a competitive advantage of MEMORIZE with respect to the uniform and threshold based baselines. The primary difference between using normalization (Figure 2) or not using normalization (Figure S7) is just a scaling factor.

[‡] The standardized mean difference (SDM) is defined as the difference in means of the treatment and control group divided by the pool standard devision of both groups.

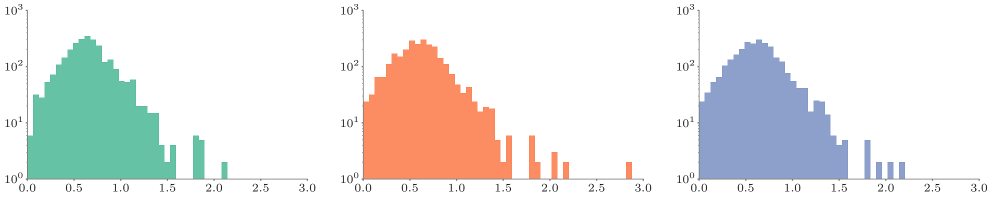

(a) MEMORIZE **(b)** Threshold **(c)** Uniform

Fig. S6. Empirical distribution of n_0 for (user, item) pairs with a training period $T = 5 \pm 0.5$ days in the treatment (MEMORIZE) and control groups (threshold based and uniform).

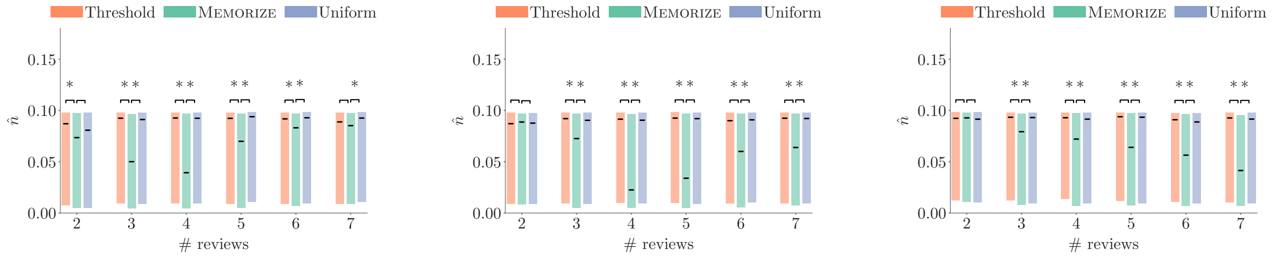

(a) $T = 3 \pm 0.3$ days **(b)** $T = 5 \pm 0.5$ days **(c)** $T = 7 \pm 0.7$ days

Fig. S7. Average empirical forgetting rate without normalization for the top 25% pairs in terms of likelihood for MEMORIZE, the uniform reviewing schedule and the threshold based reviewing schedule for sequences with different number of reviews n and different training periods $T = t_{n-1} - t_1$. Boxes indicate 25% and 75% quantiles and solid lines indicate median values, where lower values indicate better performance. MEMORIZE offers a competitive advantage with respect to the uniform and the threshold based baselines and, as the training period increases, the number of reviews under which MEMORIZE achieves the greatest competitive advantage increases. For each distinct number of reviews and training periods, $*$ indicates a statistically significant difference (Mann–Whitney U test; p-value<0.05) between MEMORIZE vs. threshold and between MEMORIZE and uniform scheduling.

References

1. F. B. Hanson. *Applied stochastic processes and control for Jump-diffusions: modeling, analysis, and computation*. SIAM, 2007.
2. John T Wixted and Shana K Carpenter. The wickelgren power law and the ebbinghaus savings function. *Psychological Science*, 18(2):133–134, 2007.
3. Harold Pashler, Nicholas Cepeda, Robert V Lindsey, Ed Vul, and Michael C Mozer. Predicting the optimal spacing of study: A multiscale context model of memory. In *Advances in Neural Information Processing Systems*, pages 1321–1329, 2009.
4. B. Settles and B. Meeder. A trainable spaced repetition model for language learning. In *ACL*, 2016.
5. Nicholas J Cepeda, Harold Pashler, Edward Vul, John T Wixted, and Doug Rohrer. Distributed practice in verbal recall tasks: A review and quantitative synthesis. *Psychological bulletin*, 132(3):354, 2006.
6. Nicholas J Cepeda, Edward Vul, Doug Rohrer, John T Wixted, and Harold Pashler. Spacing effects in learning: A temporal ridgeline of optimal retention. *Psychological science*, 19(11):1095–1102, 2008.
7. S. Leitner. *So lernt man lernen*. Herder, 1974.
8. S. Reddy, I. Labutov, S. Banerjee, and T. Joachims. Unbounded human learning: Optimal scheduling for spaced repetition. In *Proceedings of the 22nd ACM SIGKDD International Conference on Knowledge Discovery in Data Mining*, 2016.
9. Elizabeth A Stuart. Matching methods for causal inference: A review and a look forward. *Statistical science: a review journal of the Institute of Mathematical Statistics*, 25(1):1, 2010.
